# G‐quadruplex‐enhanced circular single‐stranded DNA (G4‐CSSD) adsorption of miRNA to inhibit colon cancer progression

**DOI:** 10.1002/cam4.5721

**Published:** 2023-02-28

**Authors:** Haidong Wu, Weilong Zhong, Ronghua Zhang, Yuping Ding, Chunhua Qu, Keguan Lai, Zheng Pang, Shan Yin, Guangling Zhang, Shuang Chen

**Affiliations:** ^1^ Tianjin Key Laboratory of Early Druggability Evaluation of Innovative Drugs and Tianjin Key Laboratory of Molecular Drug Research Tianjin International Joint Academy of Biomedicine Tianjin China; ^2^ Tianjin Key Laboratory of Digestive Diseases, Department of Gastroenterology and Hepatology Tianjin Institute of Digestive Diseases, Tianjin Medical University General Hospital Tianjin China; ^3^ Hebei Provincial Key Laboratory of Medical‐Industrial Integration Precision Medicine, School of Clinical Medicine North China University of Science and Technology Tangshan China; ^4^ Gastroenterology Department of Medical Center of CAPF Tianjin China; ^5^ Department of outpatient and emergency Shanghai Pudong Hospital Shanghai China; ^6^ OBiO Technology (Shanghai) Co., Ltd. Shanghai China

**Keywords:** circular single‐stranded DNA, colon cancer, G quadruplex, microRNA inhibitor, tumour suppressor gene

## Abstract

**Background:**

Chromosomal heterogeneity leads to the abnormal expression and mutation of tumor‐specific genes. Drugs targeting oncogenes have been extensively developed. However, given the random mutation of tumor suppressor genes, the development of its targeted drugs is difficult.

**Methods:**

Our early research revealed that artificial circular single‐stranded DNA (CSSD) can restore multiple tumor suppressor genes to inhibit tumor malignant progression by adsorbing miRNA. Here, we improved CSSD to a fully closed single‐stranded DNA with G quadruplex DNA secondary structure (G4‐CSSD), which made G4‐CSSD with higher acquisition rate and decreased degradation. The Cancer Genome Atlas (TCGA) and Human Protein Atlas database were used to predict tumour suppressor genes in colon cancer. Cellular and animal experiments were performed to validate the role of G4‐CSSD in cancer cell progression.

**Results:**

In colon cancer, we observed the simultaneous low expressions of chloride channel accessory 1 (CLCA1), UDP‐GlcNAc:betaGal beta‐1,3‐N‐acetylglucosaminyltransferase 6 (B3GNT6) and UDP glucuronosyltransferase family 2 member A3 (UGT2A3), which indicated an favourable prognosis. After repressing miR‐590‐3p with G4‐CSSD590, the upregulation of CLCA1, B3GNT6 and UGT2A3 inhibited the proliferation and metastasis of colon cancer cells.

**Conclusions:**

This study may provide basis for new treatment methods for colon cancer by restoration of tumor suppressor genes.

## Graphical abstract

**G4‐CSSD restorestumor suppressor genes expression to inhibit tumor progression.** CSSD with G quadruplex (G4) adsorbs microRNA like a magnet, thus releasing tumor suppressor genes and restoring their expression. So as to inhibit the progression of malignant tumor.

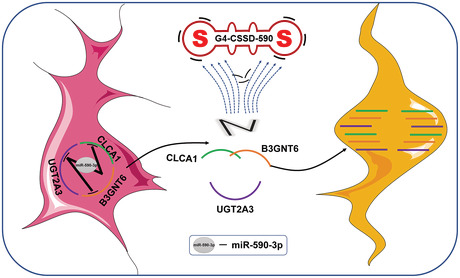

## 1 INTRODUCTION

Cancer therapy mainly includes surgical resection, chemotherapy, molecular targeted therapy and immunotherapy.^1^ Targeted immune checkpoints and CAR‐T treatments have received considerable attention in recent years.^2^ In clinical, The advanced surgical technology and postoperative treatments, such as chemotherapy and targeted therapy, play a huge role in cancer treatment. However, studies also suggested that chemotherapy‐induced drug resistance and tumour deterioration have become another problem.^3^, ^4^, ^5^ In addition, most cancer patients are unsuitable for targeted therapy due to the lack of effective drug targets.^6^ These factors limit the application of treatment methods and increase the difficulty of cancer postoperative treatment.

Drug resistance is a common and nonnegligible problem in clinical cancer therapy.^7^, ^8^ Early studies indicated that the cancer related drug resistance is driven by the combined deletion of tumour suppressor genes and abnormal elevation of oncogenes.^9^, ^10^ At present, almost all targets of cancer drugs are oncogenes or specific mutation genes in tumours but rarely tumour suppressor genes.^11^ Our earlier study revealed that artificial circular single‐stranded DNA (CSSD) can restore the expressions of multiple tumour suppressor genes, KLF17, E‐cadherin and LASS2 and inhibit the malignant progression of tumours.^12^ This finding indicates that tumours may also be cured by regulating tumour suppressor genes.

In this study, we analysed the core tumour suppressor genes that are lowly expressed in metastatic colon cancer and indicate favourable prognosis. Then, single‐stranded circular DNA with G‐quadruplex DNA secondary structure (G4‐CSSD) was used to restore the expressions of these tumour suppressor genes, thereby inhibiting the malignant progression of colon cancer. Meanwhile, G4 nucleic acid secondary structure enhanced the stability of CSSD, which will provide more support for the possibility of future drug development. Perhaps, this method will provide basis for the development of new and feasible biological therapies for the treatment of tumours from the perspective of tumour suppressor genes.

## 2 MATERIALS AND METHODS

### 2.1 Clinical data analysis

A total of 480 colon cancer cases and 41 cases of adjacent tissues in *The Cancer Genome Atlas* (TCGA) database were used to analyse genes that are under‐expressed in colon cancer and especially in metastatic tumours, which were processed by an online analysis system (https://www.xiantao.love/). In addition, 352 genes suggesting a good prognosis for colon cancer were obtained in the Human Protein Atlas database (http://www.proteinatlas.org/). Then, the core tumour suppressor genes indicating good prognosis and with low expression in metastatic colon cancer were analysed by Venn analysis (http://bioinformatics.psb.ugent.be/webtools/Venn/). Paraffin‐embedded colon cancer samples from Tianjin Medical University General Hospital were reviewed by pathologist. The study was performed in accordance with the guidelines from the Declaration of Helsinki and its amendments or comparable ethical standards.

### 2.2 G4‐CSSD design and identification

We have designed three CSSDs. Control CSSD with scramble DNA (5′‐AATTCAAAGAATTAACCTTAATTGAAGAAACAGUACUUUUGUGUAGUACAAAAACTTCAATTAAGGTTAATTCTTTG‐3′), CSSD590: miR‐590‐3p targeting CSSD without G4 loop (5′‐AATTCAAAGAATTAACCTTAATTGAAGATTACTAGCTTATACATAAAATTAAACTTCAATTAAGGTTAATTCTTTG‐3′) and G4‐CSSD590: miR‐590‐3p targeting CSSD with G4 loop (5′‐AATTCAAAGAATTAACCTTAATTGAAGGGGAGGGTTACTAGCTTATACATAAAATTAAGGGAGGGCTTCAATTAAGGTTAATTCTTTG‐3′). All the single‐stranded CSSD DNAs were synthesised by Genewiz (Beijing, China). After annealing, two DNA loops with an EcoRI site were treated with T4 DNA ligase (Takara, Beijing, China) to form a completely closed circular DNA. Both CSSD590 and G4‐CSSD590 circular DNA contained two miR‐590‐3p binding sequences. Next, G4‐CSSD590 circular DNA was annealed in a buffer of 100 mM KCl and 40% PEG200 to form circular DNA with G4 secondary DNA structure. The harvested DNA was separated and identified by native gel electrophoresis. G4 probe IZCM‐7 was donated by Prof. Jia‐Heng Tan, and the identification of G4‐CSSD was carried out in accordance with his experimental procedure.^13^

### 2.3 Cell culture and transfection

HCT116 and Caco‐2 colon cancer cells were obtained from the Cell Resource Center of the Chinese Academy of Medical Sciences. All cells were maintained in Minimum Essential Medium (MEM, Gibco, USA) containing 10% foetal bovine serum (KeyGENE, China). The full‐length coding sequences of CLCA1, BGNT6 and UGT2A3 were synthesised (Genwiz, China) and cloned into a pcDNA3.1 vector. The cells were grown for 24 h before transfection. Lipofectamine 3000 (Thermo Fisher, USA) was used to transfect the transgenic plasmid and G4‐CSSD into the cells. After 48 h, the morphological changes in the cells were detected, and the RNA and protein levels were extracted for analysis.

### 2.4 Wound healing assay

The transfected HCT‐116 and Caco‐2 cells were seeded into a 24‐well plate at 3.5 ×10^5^ cells per well. When the cell density reached 90%, the pipette tip was used to make a straight scratch. After 48 h, the migration distance was recorded under a microscope (Nikon, Japan), and the migration rate was calculated as followed: Test (0–48 h)/Ctrl (0–48 h).

### 2.5 Transwell analysis

The treated HCT‐116 and Caco‐2 cells were resuspended in serum‐free medium and seeded into the Matrigel‐coated upper chamber (Corning, USA) at a final concentration of 1 × 10^5^ per well. The complete medium containing 10% foetal bovine serum was added to the lower chamber. After 24 h, the unpassed cells were wiped off with a cotton swab, and the passed ones were fixed with 4% paraformaldehyde for 30 min. After staining with 0.1% crystal violet for 10 min and washing with running water for 2 min, the invaded cells were photographed under a microscope (Nikon, Japan).

### 2.6 Scanning electron microscope

HCT‐116 and Caco‐2 cells were seeded on a slide in 24‐well plates at a final concentration of 2 × 10^5^ per well. Transfection was performed after 24 h. After 48 h, the treated cells were fixed with 2.5% glutaraldehyde at 4°C overnight. The fixed cells were treated with different concentrations of ethanol (30%–100%) and tert‐butanol (30%–100%) and placed overnight at 4°C. After removing the tert‐butanol under vacuum and gold spraying, a scanning electron microscope (JEOL, Japan) was used to detect the cell morphology.

### 2.7 Colony formation assay

Transfected HCT‐116 and Caco‐2 cells were seeded into a six‐well plate at a density of 2000 cells per well. The cells were maintained in MEM containing 2% foetal bovine serum and continuously cultured for 10 days. The medium was changed every 3 days. When clones appeared, the medium was discarded, and the cells were fixed with 4% paraformaldehyde for 30 min. Then, the clones were stained with 0.1% crystal violet for 10 min and counted.

### 2.8 Cell viability

A cell count kit‐8 (CCK‐8 Beyotime, China) was employed to evaluate the cell viability. Cells were seeded into 96‐well plates with a density of 2000 cells per well. After 24 h, cells were transfected with the plasmid expressing miR‐590‐3p, and the empty vector was used as the control. 48 h after transfection, add 10 μL of CCK‐8 solution into each well. After incubation in the cell incubator for 2 h, the absorbance was measured at 450 nm.

### 2.9 Flow cytometry

HCT116 and Caco‐2 cells were seeded into six‐well plates at a density of 10^6^ cells per well. Cells were transfected and subsequently cultured for 48 h. After digestion with trypsin without ethylenediaminetetraacetic acid, 200 μL binding buffer was added to 10^5^ cells. Then, 5 μL Annexin V‐fluorescein isothiocyanate and 10 μL propidium iodide were added, and the cells were incubated at room temperature in the dark for 20 min. The cell apoptosis rate was analysed and measured with a flow cytometer (Millipore, USA).

### 2.10 miRNA quantitative reverse‐transcription–polymerase chain reaction (PCR)

To detect the expression of miR‐590‐3p, we used Trizol (Beyotime, China) to extract the total RNA at 48 h after transfection. A total of 5 μg RNA was used as a template, and miRNA‐specific reverse‐transcription primers were used to perform reverse‐transcription reaction to obtain the template cDNA. Then, PCR was performed using the SYBR Green qPCR Mix kit (Beyotime, China) in accordance with the manufacturer's instructions to detect the relative expression of miR‐590‐3p. U6 snRNA was used as the loading control.

### 2.11 Western blot

Cells were transfected with Lipofectamine 3000 (Thermo Fisher, USA) in accordance with the manufacturer's instructions and then cultured for 48 h. After discarding the culture medium and washing with 1 × phosphate‐buffered saline. Total cell protein was extracted with cell lysate containing 50 mM Tris (pH 7.4), 150 mM NaCl, 1% NP‐40 and 0.5% sodium deoxycholate. The quantified protein was separated by 12% sodium dodecyl sulphate‐polyacrylamide gel electrophoresis and then transferred to a polyvinylidene difluoride (PVDF) membrane (Millipore, USA). After blocking with 5% BSA for 2 h at room temperature, the PVDF membrane was incubated with primary antibodies (UGT2A3 polyclonal antibody, Thermo Fisher, 1:500; B3GNT6 polyclonal antibody, Proteintech, 1:500; CLCA1 polyclonal antibody, Affinity, 1:500) overnight at 4°C. After washing with TBST for three times, the membranes were incubated with horseradish peroxidase (HRP)‐labelled secondary antibody for 1 h at room temperature. Then, an ECL chemiluminescence kit (Beyotime, China) was used to visualise the band and detect the protein expression level in the imager (Biolight, China).

### 2.12 Animal experiment

Ten 5‐week‐old BALB/c nude mice were purchased from (Sipeifu, China). The mice were randomly divided into two groups. HCT‐116 cells were inoculated subcutaneously at an quantity of 10^7^ per mouse. G4‐CSSD was injected into the tumour 7 days after the inoculation when the tumour diameter reached 5 mm. G4‐CSSD was injected once a week, and the tumour size was measured every 3 days. The mice were euthanised by CO_2_ asphyxiation 34 days after the inoculation. The stripped solid tumours were fixed with formalin and used for immunohistochemical staining. For evaluating the role of G4‐CSSD in colon cancer liver metastasis. After the mice were anaesthetised, an incision of about 1 cm was made on the left side of the abdominal cavity and the spleen was isolated. 2 × 10^6^ HCT‐116 cells were injected into the spleen. Experimental mice were administered CSSD 20 μg/mouse every 3 days. After 5 weeks, the mice were euthanised to observe the liver metastasis. Animal studies were carried out in accordance with the Animal Use Guidelines of National Institutes for the Use of Laboratory Animals. All procedures were reviewed and approved by Animal Ethics Committee of Tianjin International Joint Academy of Biotechnology and Medicine (approval number: PD2020012).

### 2.13 Immunochemistry

The solid tumour specimens were embedded and cut into 4 μm thick sections. After deparaffinisation and washing with gradient ethanol for rehydration, the slices were placed in 3% H_2_O_2_ for 30 min to block endogenous peroxidase. The sections were then microwave boiled in citrate buffer (10 mM; pH 6.0) for antigen retrieval. Then, the sections were incubated with the following primary antibodies overnight at 4°C: UGT2A3 (1:100), B3GNT6 (1:200) and CLCA1 (1:100). The sections were then incubated with HRP‐labelled secondary antibody for 1 h at room temperature. After washing, 3,3‐diaminobenzidine staining solution (Beyotime, China) was added for 30 min. Finally, the sections were counterstained with haematoxylin and photographed under a microscope (Olympus, Japan).

### 2.14 Statistical analysis

All statistical analyses were performed using GraphPad 7.0 (version 7, GraphPad Software, Inc., La Jolla, CA, USA). Two‐tailed unpaired Student's *t*‐test was used to compare the two groups of data when normally distributed. For comparisons between multiple groups, we used analysis of variance with Bonferroni's multiple comparison analysis. Asterisks indicate significance: *, *p* < 0.05; **, *p* < 0.01; ***, *p* < 0.001.

## 3 RESULTS

### 3.1 CLCA1, UGT2A3 and B3GNT6 are simultaneously suppressed in colon cancer

**FIGURE 1:**
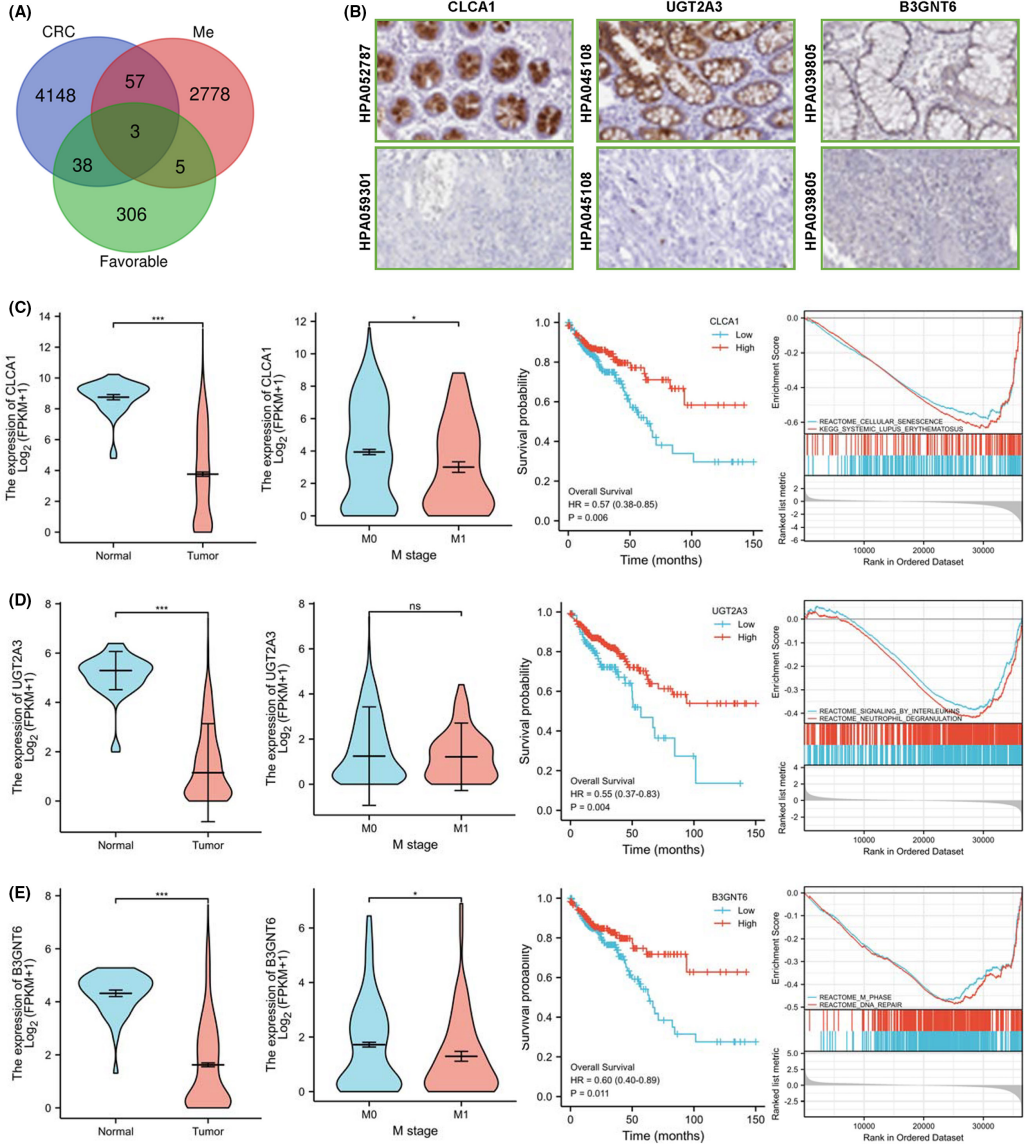
Co‐silence tumour suppressor genes in colon cancer. (A) Genes (CLCA1, B3GNT6 and UGT2A3) with low expressions in colon cancer and metastatic colon cancer were screened in the TCGA database and suggested a good prognosis. (B) Immunohistochemical staining of CLCA1, B3GNT6 and UGT2A3 in Human Protein Atlas database. (C) Expression of CLCA1 in colon cancer and metastatic tumours and its relationship with patient prognosis. (D) Expression of UGT2A3 in colon cancer and metastatic tumours and its relationship with patient prognosis. (E) Expression of B3GNT6 in colon cancer and metastatic tumours and its relationship with patient prognosis.

To obtain the core tumour suppressor genes in colon cancer, we used the TCGA and the Human Protein Atlas databases to obtain genes with low expressions in colon cancer (CRC), predict a good prognosis (favourable) and are lowly expressed in metastatic colon cancer (Me). We predicted and obtained three genes, namely, CLCA1, UGT2A3 and B3GNT6 (Figure 1A). Using the Human Protein Atlas database, we discovered that the expressions of these genes in colon cancer are lower than those in normal tissues (Figure 1B). Then, we analysed the expressions of CLCA1, UGT2A3 and B3GNT6 in 480 cases of colon cancer and 41 cases of adjacent tissues in the TCGA database and their effects on survival and metastasis. The results showed that these genes all had low expressions in tumours and had a significant negative correlation with poor prognosis (Figure 1C–E). These results suggested that CLCA1, UGT2A3 and B3GNT6 are simultaneously suppressed in the progression of colon cancer. The restoration of the expressions of these genes may inhibit the malignant progression of colon cancer.

### 3.2 Overexpression of CLCA1, UGT2A3 and B3GNT6 inhibit colon cancer cell progression

**FIGURE 2:**
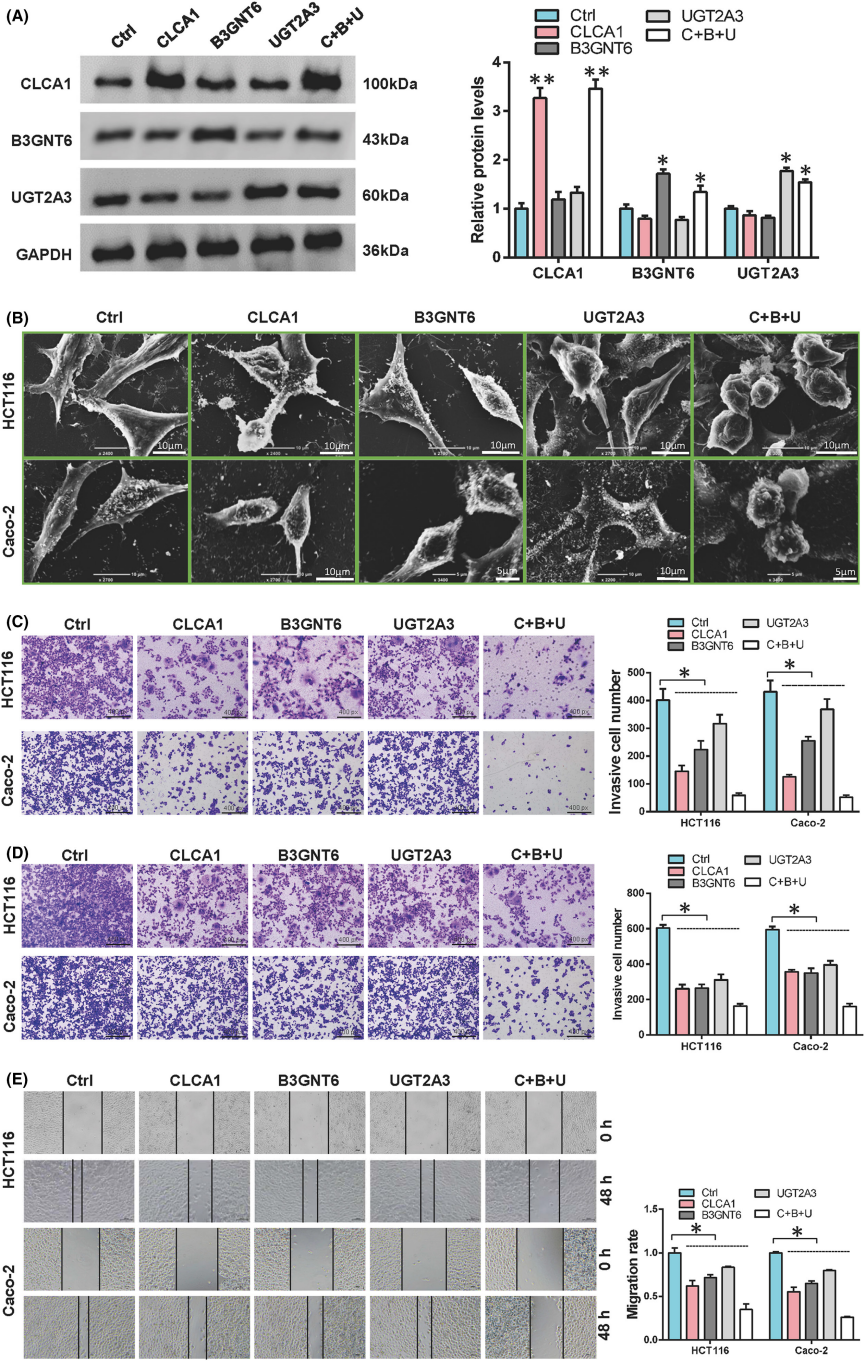
CLCA1, B3GNT6 and UGT2A3 inhibited colon cancer cell progression CLCA1, B3GNT6 and UGT2A3 were overexpressed in colon cancer cells to detect their effects on cell biological function. (A) Protein levels of CLCA1, B3GNT6 and UGT2A3 were detected by Western blot. (B) Morphological changes of cells were observed with a scanning electron microscope. (C) Cell invasive ability was analysed by transwell assay. (D) Transwell assay was used to detect cell migration. (E) Wound healing assay was performed to detect cell migration.

To verify the role of CLCA1, B3GNT6 and UGT2A3 in colon cancer, we overexpressed these genes in HCT‐116 and Caco‐2 colon cancer cells and tested their cell phenotype, proliferation, migration and invasion ability. Western blot results confirmed the effective overexpression of these genes (Figure 2A). Then, we used a scanning electron microscope to detect the cell phenotype changes. The results showed that HCT‐116 and Caco‐2 undergo apoptosis appeared after the simultaneous overexpression of CLCA1, B3GNT6 and UGT2A3 (Figure 2B). The results of transwell and wound healing assays showed that the invasion (Figure 2C) and migration ability (Figure 2D,E) was inhibited after the overexpression of CLCA1, B3GNT6 and UGT2A3. These results indicated that the expressions of CLCA1, B3GNT6 and UGT2A3 can inhibit the malignant progression of colon cancer cells.

### 3.3 CLCA1, UGT2A3 and B3GNT6 were regulated by miR‐590‐3p

**FIGURE 3:**
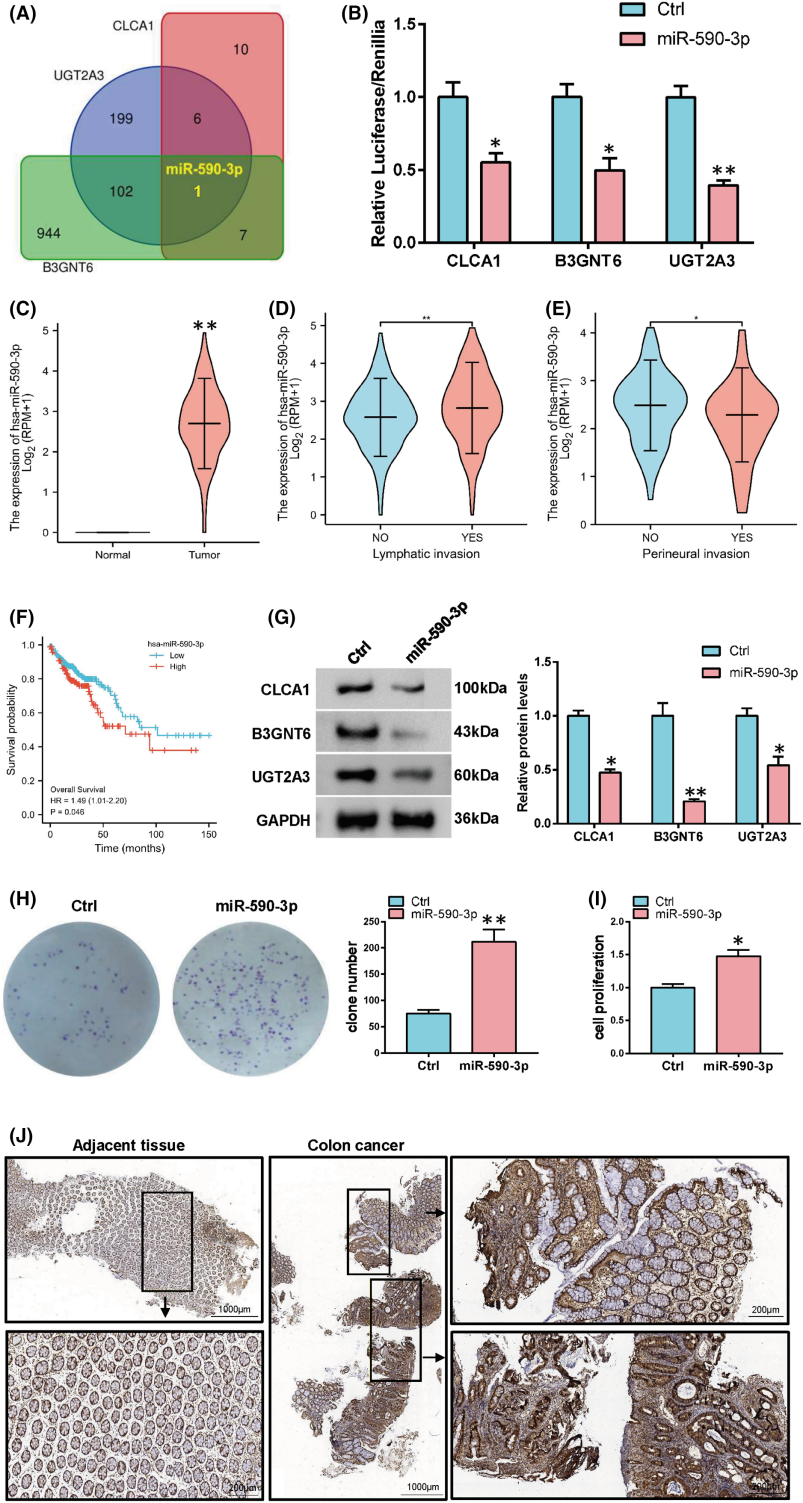
miR‐590‐3p simultaneously targets CLCA1, B3GNT6 and UGT2A3. (A) TargetScan was used to predict that miR‐590‐3p simultaneously regulates CLCA1, B3GNT6 and UGT2A3. (B) miR‐590‐3p can bind to the 3′ UTR of CLCA1, B3GNT6 and UGT2A3 by luciferase reporter assay. (C) mRNA levels of miR‐590‐3p in colon cancer and normal tissues. (D and E) mRNA levels of miR‐590‐3p in metastatic and non‐metastatic colon cancer. (F) Colon cancer patients with high expression of miR‐590‐3p in the TCGA database suggest a poor prognosis. (G) Western blot was performed to detect the protein levels of CLCA1, B3GNT6 and UGT2A3 after miR‐590‐3p overexpression in Caco‐2 cells. (H) CCK8 assay was performed to detect the role of miR‐590‐3p on cell viability. (I) Cell proliferation was evaluated by colony formation assay. (J) miR‐590‐3p expression detected in clinical colon cancer tissues.

To verify the upstream miRNAs that can simultaneously regulate CLCA1, B3GNT6 and UGT2A3, we used the TargetScan database to observe that only miR‐590‐3p can simultaneously bind to the mRNA of CLCA1, B3GNT6 and UGT2A3 (Figure 3A). Then, we cloned the wild‐type 3′ untranslated region (UTR) sequences of CLCA1, B3GNT6 and UGT2A3 into the firefly luciferase reporter vector. After co‐transfecting the luciferase reporter vector with miR‐590‐3p into HCT116 cells, miR‐590‐3p inhibited the activity of luciferase‐carrying wild‐type 3′ UTR (Figure 3B). Next, we used the TCGA database analysis and observed that miR‐590‐3p was highly expressed in colon cancer and associated with metastasis (Figure 3C–E). In addition, the high expression of miR590‐3p in colon cancer indicated poor prognosis (Figure 3F). Western blot analysis indicated that the overexpression of miR‐590‐3p can reduce the protein levels of CLCA1, B3GNT6 and UGT2A3 (Figure 3G). CCK8 and colony formation assay were performed to detect the effect of miR‐590‐3p overexpression on cell proliferation. Results showed that miR‐590‐3p could promote cell proliferation (Figure 3H,I). We also detected the expression of miR‐590‐3p in colon cancer by in situ hybridisation. The results indicated that miR‐590‐5p was highly expressed in colon cancer compared with normal tissues (Figure 3J). This result indicates that miR‐590‐3p may promote the malignant progression of colon cancer cells by regulating the expression of CLCA1, B3GNT6 and UGT2A3.

### 3.4 G4‐CSSD590 can adsorb miR‐590‐3p stably and effectively

**FIGURE 4:**
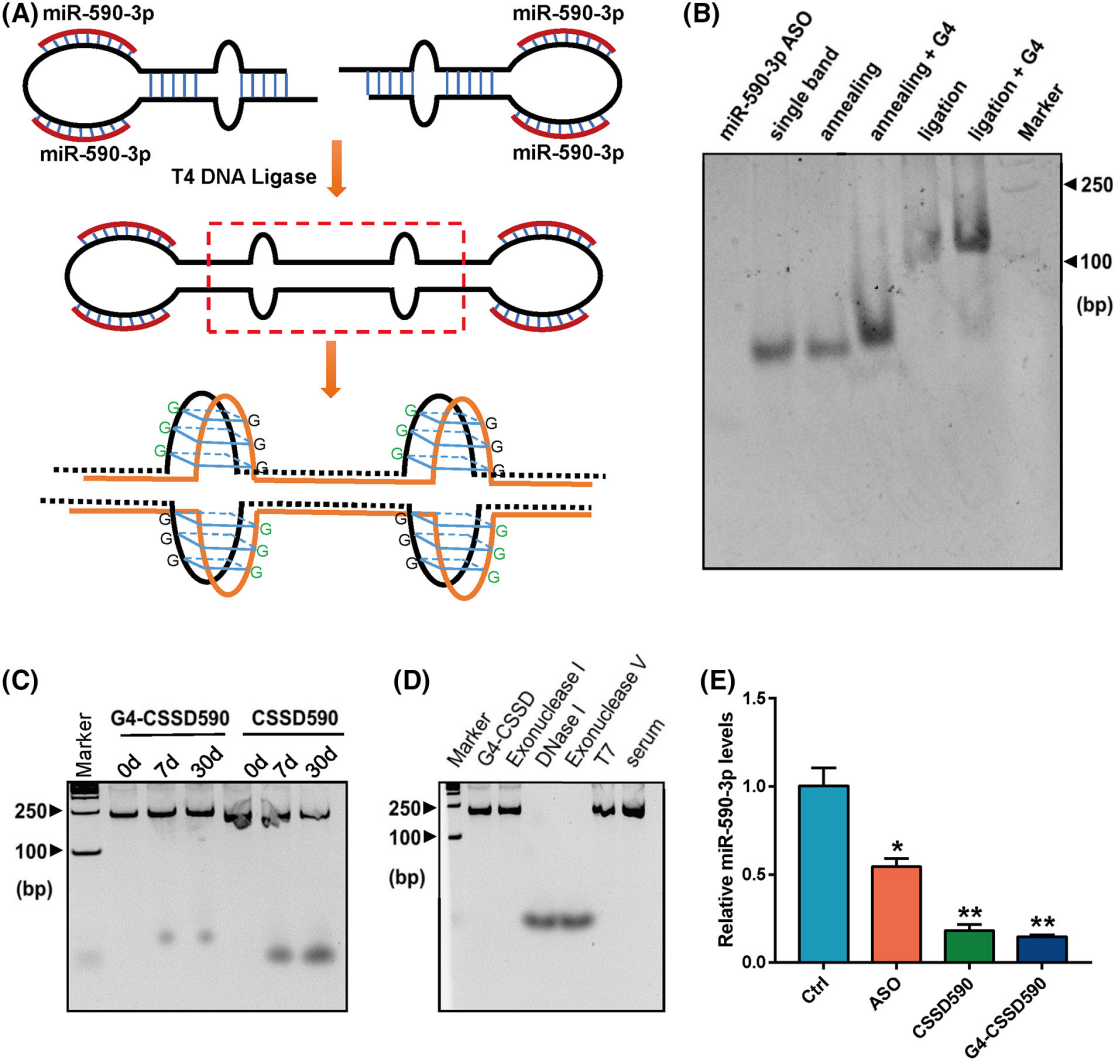
G4 secondary DNA structure can enhance the stability of circular single‐stranded DNA (CSSD). (A) Design route of G4‐CSSD. (B) G4 probe (IZCM‐7) was used to detect G4‐CSSD separated by native gel electrophoresis. miR‐590‐3p ASO (miR‐590‐3p antisense oligo), single band (unannealed single‐stranded G4‐CSSD590), annealing (annealing G4‐CSSD590), annealing+G4 (annealed G4‐CSSD590 in buffer containing K+ and PEG200 induced the formation of G4), ligation (annealed G4‐CSSD590 forms a dimeric closed structure under the action of T4 ligase) ligation+G4 (Dimeric G4‐CSSD590 was induced to form a G4 structure in a buffer containing K+ and PEG200). (C) G4‐CSSD590 and CSSD590 stored at 4°C for different times were examined using native gel electrophoresis. (D) G4‐CSSD590 treated with nuclease and serum. (E) miR‐590‐3p levels in HCT116 cells after CSSD590 or G4‐CSSD590 treatment.

In our early research, we designed CSSD to simultaneously upregulate the expressions of multiple tumour suppressor genes by adsorbing miR‐9. Here, we used G4 DNA secondary structure to increase the stability of CSSD. The single‐stranded DNA was annealed to form a secondary DNA structure containing miR‐590‐3p antisense nucleic acid sequence, multiple triple guanylic acids and sticky ends. After forming a complete closed‐loop structure under the action of T4 DNA ligase, G4‐CSSD590 was induced to form in a buffer containing K+ and PEG200 (Figure 4A). Then, miR‐590‐3p antisense oligo, single band (unannealed single‐stranded G4‐CSSD590), annealing G4‐CSSD590, annealing+G4 (annealed G4‐CSSD590 in buffer containing K+ and PEG200 induced the formation of G4), ligation (annealed G4‐CSSD590 forms a dimeric closed structure under the action of T4 ligase) ligation+G4 (Dimeric G4‐CSSD590 was induced to form a G4 structure in a buffer containing K+ and PEG200). These DNAs were separated by native polyacrylamide gel electrophoresis and stained with IZCM‐7. The results showed that ASO did not form the secondary structure of G4, but the nucleic acid with multiple consecutive guanines formed the secondary structure of G4 DNA without induction, although in a smaller amount. (Figure 4B). We compared the degradation of CSSD590 and G4‐CSSD590 at room temperature. CSSD590 was degraded after 7 days, while no significant degradation was found for G4‐CSSD590 (Figure 4C). After treating G4‐CSSD590 with a variety of exonucleases and endonucleases, no visible degradation fragments were found (Figure 4D). Thus, the stability of G4‐CSSD590 was enhanced by G4 secondary structure. Then, CSSD590 and G4‐CSSD590 were transfected into HCT116 cells. The PCR results showed that G4‐CSSD590 can adsorb miR‐590‐3p and lead to a significant downregulation of miR‐590‐3p expression (Figure 4E).

### 3.5 G4‐CSSD590 inhibits the proliferation and invasion of colon cancer cells

**FIGURE 5:**
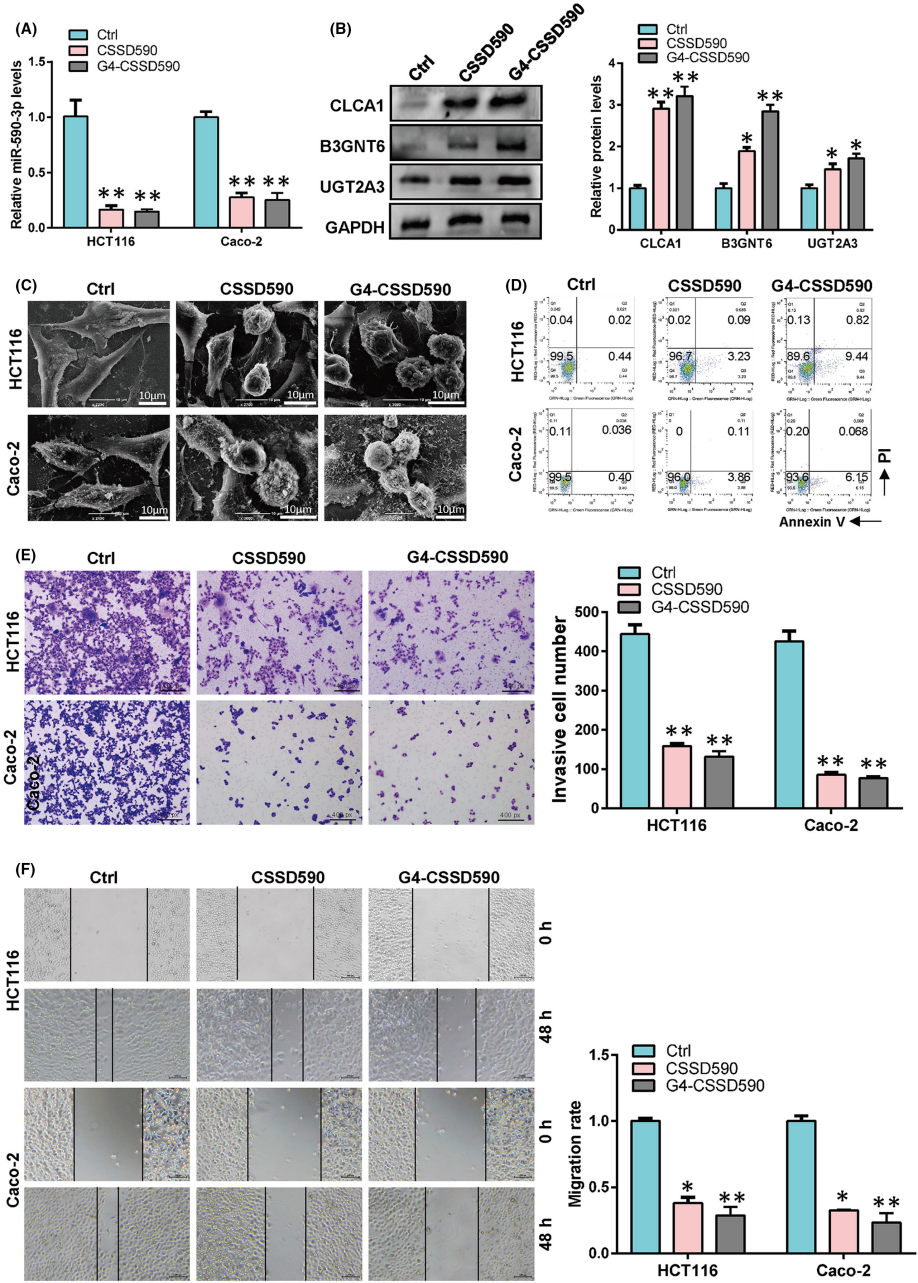
G4‐CSSD590 suppressed colon cancer cell progression. Caco‐2 and HCT116 cells were treated with Ctrl CSSD, CSSD590 and G4‐CSSD590 to downregulate miR‐590‐3p and release CLCA1, B3GNT6 and UGT2A3. (A) miR‐590‐3p RNA levels in Caco‐2 and HCT116 cells after G4‐CSSD‐590 transfection. (B) Western blot was used to examine the protein levels of CLCA1, B3GNT6 and UGT2A3. (C) Scanning electron microscopy was used to examine the effect of G4‐CSSD‐590 on cell phenotype. (D) Apoptosis was detected by flow cytometry. (E and F) Cell invasion and migration abilities were examined by transwell and wound healing assays.

To confirm whether G4‐CSSD590 can restore the expression of CLCA1, UGT2A3 and B3GNT6 and its role in colon cancer after the adsorption of miR‐590‐3p, we transfected Ctrl CSSD, CSSD590 and G4‐CSSD590 in HCT116 and Caco‐2 cells. PCR results indicated that both CSSD590 and G4‐CSSD590 can effectively reduce the level of miR‐590‐3p (Figure 5A). The decreasing of miR‐590‐3p led to the upregulation of CLCA1, UGT2A3 and B3GNT6 protein levels (Figure 5B). Scanning electron microscopy showed that colon cancer cells underwent apoptosis after CSSD590 treatment, whereas apoptosis appeared to be more severe in G4‐CSSD590‐treated cells (Figure 5C). Meanwhile, we used flow cytometry to verify that CSSD590 and G4‐CSSD590 can promote the apoptosis of colon cancer cells (Figure 5D). In addition, both CSSD‐590 and G4‐CSSD590 inhibited the invasion (Figure 5E) and migration (Figure 5F) of colon cancer cells (Figure 5E,F). These results indicated that both G4‐CSSD590 and CSSD590 could restore the expression of multiple tumour suppressor genes and inhibit the malignant progression of colon cancer cells.

### 3.6 G4‐CSSD590 inhibits colon cancer tumour growth by restoring the expressions of CLCA1, UGT2A3 and B3GNT6

**FIGURE 6:**
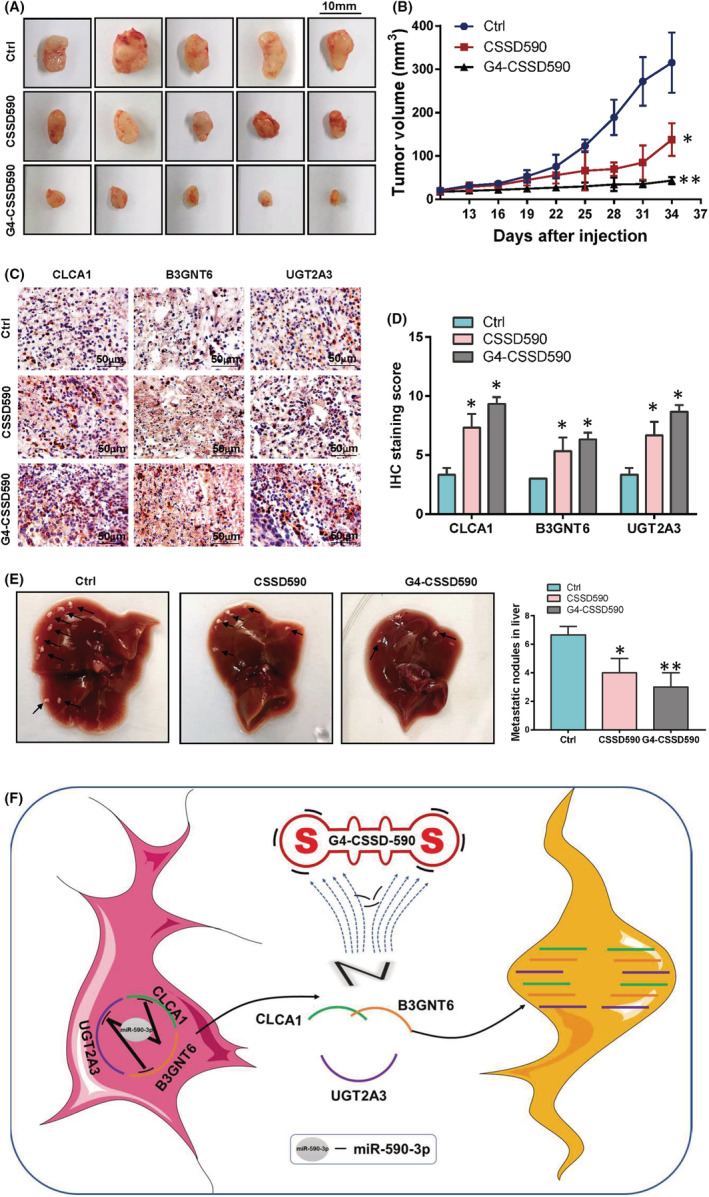
G4‐CSSD‐590‐3p inhibited tumour growth and liver metastasis of colon cancer. (A) Solid tumours of subcutaneously inoculated colon cancer cells after CSSD590 and G4‐CSSD‐590 treatment. (B) Solid tumours of subcutaneously inoculated colon cancer cells. (C and D) Immunohistochemistry was used to detect the expressions of CLCA1, B3GNT6 and UGT2A3 in solid tumour tissues. (E) Liver metastases after spleen inoculation of colon cancer cells with G4‐CSSD‐590 intervention. (F) Graphic abstract.

5‐week‐old female BALB/c nude mice were randomly divided into three groups. HCT‐116 cells were inoculated subcutaneously with the total amount of 10^6^ per mouse. When the diameter of the solid tumour reached 5 mm 10 days after the inoculation, Ctrl CSSD, CSSD590 and G4‐CSSD590 were injected into solid tumours at 20 μg/mouse. CSSDs were injected on Days 10, 17 and 24. The tumour size was measured and recorded every 3 days. The mice were euthanised 10 days after the last injection. After the measurement of tumour size, the solid tumour tissue was fixed in formalin solution to detect the expressions of CLCA1, UGT2A3 and B3GNT6. Experimental results showed that G4‐CSSD can inhibit tumour growth (Figure 6A). The tumour growth curve illustrated the difference in tumour growth after CSSD590 and G4‐CSSD590 treatment. Here, the tumour suppressor effect of G4‐CSSD590 was stronger than that of CSSD590. We speculate that it may be due to the ability of the G4 secondary structure to resist degradation. (Figure 6B). The results of immunohistochemistry experiments showed that the expressions of CLCA1, UGT2A3 and B3GNT6 in solid tumours were all upregulated after CSSD590 and G4‐CSSD590 treatment (Figure 6C,D). In addition, G4‐CSSD590 can effectively inhibit liver metastasis of colon cancer cells (Figure 6E). The meaning graphic abstract is tumour suppressor genes cannot exert their roles after being inhibited by miRNAs. After G4‐CSSD adsorbs miRNA like a magnet, tumour suppressor genes were released and exert tumour suppressor function (Figure 6F).

## 4 DISCUSSION

Chemotherapy, including postoperative chemotherapy and preoperative neoadjuvant chemotherapy, is indispensable in cancer treatment.^14^ While killing tumour cells, several chemotherapeutic drugs may also promote tumour cell metastasis.^15^, ^16^, ^17^ The malignant progression and metastasis of tumours caused by chemotherapy may be caused by the effects of drugs on the tumour microenvironment and the stress response of tumour cells to drugs.^18^, ^19^ Meanwhile, chemotherapy lacks a specific target and threatens the survival of normal cells while killing tumour cells.^20^, ^21^ Therefore, a combination of chemical drugs and targeted drugs is used clinically.^22^, ^23^ However, with the evolution of tumour cells, targeted drugs may also lose their therapeutic effects at variable times.

Postoperative chemotherapy drugs fluoropyrimidine and oxaliplatin reduce the risk of colon cancer tumour recurrence.^24^, ^25^ The application of targeted drugs, such as panitumumab, has also shown efficacy in patients who are intolerant to chemotherapy. However, the efficacy of these drugs is related to activating mutations in KRAS.^26^ The most common changes in colorectal cancer also include APC, BRAF, SMAD4, TP53, PIK3CA and so on.^27^, ^28^ Targeted drugs for a single mutation site of oncogenes hardly meet the needs of clinical treatment. The development of drugs targeting tumour suppressor gene mutations is more difficult to achieve because mutations at any site may cause tumour suppressor genes to lose their function.^29^ Here, we did not consider mutated tumour suppressor genes but obtained multiple tumour suppressor genes with low expressions in metastatic colon cancer and suggest a good prognosis. We found three candidate core, tumour, suppressor genes in colon cancer, namely, CLCA1, B3GNT6 and UGT2A3. CLCA1 as a chloride channel regulator has been confirmed to be related to a variety of cell functions. N‐Acetylglucosamine is responsible for creating the core structure of O‐glycans, which are an important part of mucin‐type glycoproteins. The O‐glycan in the mucin core protein is the main component of intestinal mucus and constitutes a part of the intestinal mucosal barrier. C3GnT‐deficient mice have increased intestinal barrier permeability and are likely to induce colitis and colorectal adenocarcinoma.^30^ This condition suggests that in addition to abnormal gene expression, the occurrence and progression of colon cancer are closely related to the intestinal barrier, metabolism and microenvironment.

MicroRNAs (miRNAs) are a class of short non‐coding RNA molecules that lead to mRNA translational inhibition or degradation at post‐transcriptional level. Functional studies have confirmed that miRNA dysregulation is a causal relationship in many diseases, especially cancer.^31^, ^32^ MicroRNA (miRNA)‐based therapeutics can be divided into miRNA mimics and miRNA inhibitors (also known as antimiRs). antimiRs include antisense oligonucleotides (ASOs) and miRNA‐sponge. So far, the most popular antimiR is still chemical modified ASO, such as locked‐nucleotide, 2ʹ‐O‐methoxyethyl modification.^33^ These modifications could increase the biological stability of ASO. However, the single‐stranded nucleic acid still degrades to some extent. The circular nucleic acid structure can avoid the degradation of nuclease and increase the bioavailability of antimiR. The G4 secondary DNA structure consists of two or more stacked guanine tetrads. G4s can play a regulatory role in DNA replication and transcription, especially oncogenes. In addition, the G4 secondary DNA structure contained in the BCL‐2 promoter is closely related to the transcriptional regulation of the BCL‐2 gene.^34^, ^35^ The promoter region of the genomic DNA of KRAS, which is frequently mutated in tumours, also contains the G4 structure.^36^ During the transcription process, transcription factors, such as SP1 and RNA Pol II, can recognise and bind G4 DNA to regulate gene expression. The G4 interactive small molecule, NSC 82892, combined with the G4 of the vascular endothelial growth factor (VEGF) promoter can prevent the recruitment of SP1 and RNA Pol II and thus inhibit VEGF transcription.^37^, ^38^ Accumulated evidence has shown that the G4 secondary DNA structure has good stability and is widely involved in the regulation of gene expression. Therefore, we introduced the G4 structure into CSSD to increase the stability of CSSD. The results showed that CSSD containing G4 structure had a longer half‐life and did not affect the adsorption efficiency of miRNA.

In summary, we have discovered that CLCA1, B3GNT6 and UGT2A3 were lowly expressed in metastatic colon cancer and predicted poor prognosis. The use of G4‐CSSD to restore the expression of these genes by adsorbing miR‐590‐3p can inhibit the malignant progression of colon cancer cells. Meanwhile, G4 secondary nucleic acid results can increase the biological stability of CSSD. These results suggest that innate immunity and tumour microenvironment play an important role in colon cancer. In addition, nucleic acid drug molecules that can suppress tumours by simultaneously restoring multiple key tumour suppressor genes may be proposed.

## AUTHOR CONTRIBUTIONS

**Haidong Wu:** Conceptualization (equal); data curation (equal); investigation (equal); methodology (equal); writing – original draft (equal). **Weilong Zhong:** Conceptualization (supporting); investigation (equal); methodology (equal); project administration (equal); writing – original draft (equal). **Ronghua Zhang:** Investigation (equal); project administration (equal); software (equal); visualization (equal). **Yuping Ding:** Investigation (equal); resources (equal); writing – original draft (equal). **Chunhua Qu:** Methodology (equal); validation (equal); writing – original draft (equal). **Keguan Lai:** Data curation (equal); software (equal); validation (equal). **Zheng Pang:** Investigation (equal); methodology (equal); software (equal); visualization (equal). **Shan Yin:** Methodology (equal); resources (equal); visualization (equal). **Guangling Zhang:** Conceptualization (equal); funding acquisition (equal); supervision (equal); validation (equal); writing – original draft (lead); writing – review and editing (equal). **Shuang Chen:** Conceptualization (lead); data curation (equal); writing – review and editing (equal).

## ETHICS STATEMENT

All animal experiments were reviewed and approved by Animal Ethics Committee of Tianjin International Joint Academy of Biotechnology and Medicine.

## Data Availability

All data can be obtained by contacting corresponding author.
